# Psychometric properties of an Arabic translation of the multidimensional social support scale (MSPSS) in a community sample of adults

**DOI:** 10.1186/s12888-023-04937-z

**Published:** 2023-06-14

**Authors:** Feten Fekih-Romdhane, Mirna Fawaz, Rabih Hallit, Toni Sawma, Sahar Obeid, Souheil Hallit

**Affiliations:** 1 The Tunisian Center of Early Intervention in Psychosis, Department of Psychiatry “Ibn Omrane”, Razi hospital, Manouba, 2010 Tunisia; 2 Faculty of Medicine of Tunis, Tunis El Manar University, Tunis, Tunisia; 3 Faculty of Health Sciences, Beirut Arab University, Tareek Al Jadida, Afeef Al Tiba, Beirut, 1105 Lebanon; 4 School of Medicine and Medical Sciences, Holy Spirit University of Kaslik, P.O. Box 446, Jounieh, Lebanon; 5 Department of Infectious Disease, Bellevue Medical Center, Mansourieh, Lebanon; 6 Department of Infectious Disease, Notre Dame des Secours University Hospital, Postal code 3, Byblos, Lebanon; 7 School of Arts and Sciences, Social and Education Sciences Department, Lebanese American University, Jbeil, Lebanon; 8 Psychology Department, College of Humanities, Effat University, Jeddah, 21478 Saudi Arabia; 9 Applied Science Research Center, Applied Science Private University, Amman, Jordan; 10 Research Department, Psychiatric Hospital of the Cross, Jal Eddib, Lebanon

**Keywords:** Social support, Confirmatory factor analysis, Psychometric properties, Arabic

## Abstract

**Background:**

There is a lack of methodologically strong measure to assess perceived social support among Arabic-speaking populations. Our main objective was therefore to examine the psychometric properties of an Arabic translation of the Multidimensional Social Support Scale (MSPSS) in a sample of Arabic-speaking Lebanese adults from the general population.

**Methods:**

We adopted a cross-sectional design involving a convenience sample of 387 non-clinical Lebanese adults aged 26.17 ± 11.47 years (58.4% females). Participants were administered a web-based anonymous questionnaire containing the MSPSS, the 10-item Connor-Davidson Resilience Scale, and the Post traumatic growth Inventory-Short Form. The forward-backward translation method was applied. Confirmatory factor analysis (CFA) and gender invariance in the MSPSS were examined. McDonald’s ω coefficients were calculated as internal consistency indicators.

**Results:**

The Arabic MSPSS and its subscales have a high internal consistency with McDonald’s ω values between 0.94 and 0.97. CFA indicated that fit of the three-factor model was acceptable. All indices suggested that configural, metric, and scalar invariance was supported across gender. Both genders exhibited no significant difference in all MSPSS dimensions. Convergent validity was supported by showing that all three MSPSS sub-scores and total score correlated significantly and positively with resilience and posttraumatic growth scores.

**Conclusion:**

Although further cross-cultural validations involving other Arab countries and communities are still needed, we preliminarily suggest that this scale is applicable to the broad Arabic-speaking people for the measurement of perceived social support in clinical and research contexts.

## Introduction

Social support is a multifaceted and complex concept that refers to the amount of assistance an individual can get through interpersonal interactions [1, 2], including family, friends, peers, and members of a community [3]. As opposed to received social support (i.e., the actual support that one receives), perceived social support infers the beliefs and/or perceptions of the already-present support provided by the social network when needed [4–7]. Social support has an important role in human health [8]. Its adequate availability seems essential to provide a buffer for stressful physical and psychosocial events through greater resilience [9, 10], promote self-esteem [11], and mitigate the effects of psychological distress [12]. In this regard, perceived social support has been argued to have a more significant impact on health determinants compared to actual received social support [13, 14]. However, the existing literature on the effect of social support on physical, mental health and quality of life has led to mixed findings; which is partly due to the use of different measurement instruments [15].

A number of social support measures have been developed and tested in various groups and populations [16]. Out of these measures, the Multidimensional Scale of Perceived Social Support (MSPSS) is one of the most widely used worldwide [3, 6–8, 11, 12, 18]. The MSPSS is a 12-item brief, freely available, easy to administer, self-report scale designed by Zimet et al. to subjectively assess “the adequacy of received emotional social support” from three different sources (family, friends, and the significant other) ([17], p. 186). The original English version of the MSPSS consists of a three-factor construct which had high internal consistency and test-retest reliability, as well as construct validity [17]. The scale has been translated in many languages (e.g., Italian [18], Swedish [19], Polish [20], Portuguese [21], Greek [22], South Korean [23], Turkish [24], Persian [25], Indian [26], Urdu [27], Thai [28], Hausa (Nigerian) [29], Ugandan [30], Malawi [31], Malay [32]) and countries (e.g., high- [19, 21–23], middle- [24, 25, 28, 32, 33], and low-income countries [29–31]) across the globe. The psychometric properties of the MSPSS have proven their appropriateness in individuals from a variety of cultural backgrounds, ages, and clinical profiles. For instance, the Swedish version of the MSPSS reproduced the original three-factor structure and supported the good validity and internal consistency (α = 0.91–0.95) of the scale in samples of women with hirsutism and nursing students [19]. The Polish validation also supported the factorial validity of the MSPSS among university students, as well as good reliability (α = 0.89-0.94) and concurrent validity as evidenced through adequate patterns of associations with psychological indicators (anxiety, loneliness, life satisfaction and current involvement in a romantic relationship) [20]. Several other translated versions of the MSPSS provided support to the expected three-factor structure, and showed strong internal consistency, including Malawi [31], Nigerian Hausa [34], Thai [35], and Spanish [36]. Tonsing et al. [37] found that the Nepali translation of the MSPSS confirmed the original three-factor model, whereas the Urdu version could retain only two factors, with the subscales of Significant Others and Family being combined into one factor and cultural factors suggested to partly explain these discrepancies. Both versions revealed high internal reliability and construct validity in two samples of South Asians living in Hong Kong [37]. Measurement invariance across gender groups was verified and established in different MSPSS linguistic versions and populations, including and Chinese parents of children with cerebral palsy [38], Spanish patients with cancer [36], Romanian elite athletes [39], as well as Chinese [40], Nigerian [41] Romanian [42], and Indonesian adolescents [43].

While there is evidence asserting the psychometric strength of the MSPSS across different contexts [44], literature has also documented a substantial impact of culture on social support access and sources [45–47]. For instance, collectivist cultures promote social cohesion and parenting/family relationships quality; therefore, individuals from such cultures expect extended family than other sources to supply them with any needed social support [48]. Despite these data, the largest amount of research on social support has emerged from the Western/Eastern world. In addition, the original validation study and subsequent studies examining the MSPSS psychometric quality have been mostly performed in these cultural backgrounds. This limits our knowledge about the pathways linking social support to mental health and prevents evidence-based policymaking in the under-researched contexts, including Arab countries and communities from the Middle East and North Africa (MENA) region.

There are a total of 22 Arab countries geographically distributed over two continents (i.e., Africa and Asia), most of them defined as lower-middle-income economies, traditional, religious and collectivist societies [49, 50], and having a current population estimated at greater than 450 million people [51]. Arabic is thus spoken by hundreds of millions of people in both Arab and non-Arab countries. Over the last decades, Arab countries have faced a series of revolutions, armed conflicts, terrorist attacks, widespread violence, traumatic wars, and economic recessions, which have negatively affected their local communities’ mental health [52–54]. At the same time, Arab countries suffer a substantial lack of information, mental health legislation and policy [54]. One of the main factors that impede access to evidence-informed care and mental health research in Arab countries is the shortage of valid and reliable assessment tools [55]. As we specifically focus on perceived social support in the present study, we point to the little information available on this construct in Arab contexts. We could find only a few studies among Arab people using the MSPSS in specific populations (e.g., Arab American adolescents [56] and women [57], Arab immigrant women [58], refugees in Jordan [59], mothers of children with developmental disabilities [60]); which are far from being representative of the Arab general population. All these observations highlight the strong need for an Arabic valid tool to evaluate social support.

A systematic review published in 2018 by Dambi et al. [61] investigated the psychometric properties of the non-English translations of the MSPSS found only one Arabic version available (i.e., [33]). The authors described its methodology as “poor” based on poor internal consistency and validity (no confirmatory factor analysis performed). Dambi et al. [61] also estimated that this version had “unknown evidence for construct validity” and provided “scanty details” for the adaptation process; which may in turn lead to the risk of misleading findings and negatively affect policy formulation. These potential methodological flaws encouraged our team to translate and validate the MSPSS to the Arabic language, in order to address the identified gaps of the previous Arabic version and provide a psychometrically sound social support scale for the Arabic-speaking researchers, clinicians, patients and the broad community. Our main objective was therefore to examine the psychometric properties of an Arabic translation of the MSPSS in a sample of Arabic-speaking Lebanese adults from the general population. We expect that the Arabic MSPSS will (1) replicate the original three-factor structure; and (2) yield good internal consistency, convergent validity, and measurement invariance across gender groups. We expected to demonstrate measurement invariance at the configural, metric, and scalar levels. Given that strict invariance is very hard to meet, and thus rarely hold [62]; and since this form of measurement invariance is acknowledged to be overly restrictive [63], we did not expect to be capable to show invariance at this level. Convergent validity was tested through demonstrating that MSPSS scores correlate to other relevant constructs (here, resilience and posttraumatic growth) in the theoretically expected way [64]. We chose these correlates because social support has consistently been demonstrated to promote behaviours that improve stress-regulation, such as enhancing resilience and growth [65]. Indeed, a strong evidence exists supporting that social support is a potential attribute of resilience [66] and post-traumatic growth [67, 68]. We thus expect to establish convergent validity of the MSPSS by demonstrating positive correlations between social support and both resilience and post-traumatic growth scores.

## Methods

### Translation & adaptation procedures

Before their use in the current study, the MSPSS scale was translated and adapted to the Arabic language and context. To this end, it was translated to the Arabic language with the purpose of achieving semantic equivalence between measures in their original and Arabic versions following international norms and recommendations [69]. For this, the forward and backward translation method was applied. The English version was translated to Arabic by a Lebanese translator who was completely unrelated to the study. Afterwards, a Lebanese psychologist with a full working proficiency in English, translated the Arabic version back to English. The translation team ensured that any specific and/or literal translation was balanced. The initial and translated English versions were compared to detect/eliminate any inconsistencies and guarantee the accuracy of the translation by a committee of experts composed of two psychiatrists and one psychologist, in addition to the research team and the two translators [70]. An adaptation of the measure to our specific context was performed, and sought to determine any misunderstanding of the items wording as well as the ease of items interpretation; and, therefore, ensure the conceptual equivalence of the original and Arabic scales in both contexts [71]. After the translation and adaptation of the scale, a pilot study was done on 20 patients to ensure all questions were well understood; no changes were applied after the pilot study.

### Measures

#### The multidimensional scale of perceived social support (MSPSS)

It is a succinct research instrument, gauging the degree of individual perceptions of social support that emanates from three distinct sources: Family, Friends and a Significant Other—measured by three subscales of four items each, with a total number of items of 12. Each item is rated on a 7-point Likert rating scale (1 = Very strongly Disagree, 7 = Very strongly agree). Higher scores express stronger feelings of being socially supported [17]. The original scale yielded good psychometric properties, with Cronbach’s alpha coefficients ranging from 0.85 to 0.91 for total the total score and three sub-scores. We used the Arabic version already translated by Merhi and Kazarian [33].

#### The 10-item connor-davidson resilience scale (CD-RISC-10)

The CD-RISC-10 comprises a single factor of 10 items [72, 73], each of which are scored on a 5-point scale ranging from 0 (not true at all) to 4 (true nearly all of the time). Examples of items include, “I am able to adapt when changes occur” and “I am not easily discouraged by failure.” Higher scores on the CD-RISC-10 indicate higher levels of resilience. The original English version displayed excellent psychometric properties (Cronbach’s alpha = 0.85 for total scores). In the present sample, the McDonald’s ω value was 0.89.

#### Post traumatic growth inventory-short form (PTGI-SF)

This scale measures favourable outcomes after a traumatic event, including 5 dimensions: relating to others, new possibilities, personal strength, spiritual change and appreciation of life. The 10-item scale was used in this study [74]. Each item is scored on a 6-point Likert type scale ranging from 0 (“I did not experience this change as a result of my crisis”) to 5 (“I experienced this change to a very great degree as a result of my crisis”). The original PTGI-SF showed appropriate internal consistency (α = 0.86 to 0.89) [75, 76]. Our sample yielded a McDonald’s ω value of 0.95. The Arabic versions of the CD-RISC-10 and PTGI-SF are already validated in Arabic [77].

#### Demographics

Participants were asked to provide their demographic details consisting of age, sex, highest educational attainment, region of living, and marital status.

### Procedures

All data were collected via a Google Forms link; the sample was recruited conveniently between May and July 2022. The project was advertised on social media and included an estimated duration. Inclusion criteria for participation included: (1) being of a resident and citizen of Lebanon, (2) aged 18 years and above, (3) having access to the Internet, and (4) willing to participate in the study. Excluded were those who refused to fill out the questionnaire. Internet protocol (IP) addresses were examined to ensure that no participant took the survey more than once. After providing digital informed consent, participants were asked to complete the instruments described above, which were presented in a pre-randomised order to control for order effects. The survey was anonymous and participants completed the survey voluntarily and without remuneration [78].

### Analytic Strategy

#### Confirmatory factor analysis (CFA)

There were no missing responses in the dataset. We used data from the total sample to conduct a CFA using the SPSS AMOS v.26 software. As a rule of thumb, simulation studies show that with normally distributed indicator variables and no missing data, a reasonable sample size for a simple confirmatory factor analysis model is about N = 150 [79], which was exceeded in our sample. Our intention was to test the original model of the MSPSS scores (i.e., three-factor model). Parameter estimates were obtained using the maximum likelihood method and fit indices. For this purpose, the normed model chi-square (χ²/df), the Steiger-Lind root mean square error of approximation (RMSEA), the Tucker-Lewis Index (TLI) and the comparative fit index (CFI). Values ≤ 5 for χ²/df, and ≤ 0.08 for RMSEA, and 0.90 for CFI and TLI indicate good fit of the model to the data [80?]. Additionally, evidence of convergent validity was assessed in this subsample using the Fornell-Larcker criterion, with average variance extracted (AVE) values of ≥ 0.50 considered adequate [81?] and meaning that a latent variable is able to explain more than half of the variance of its indicators on average (i.e., items converge into a uniform construct). The absence of multicollinearity was verified through tolerance values > 0.2 and variance inflation factor (VIF) values < 5. Multivariate normality was not verified at first (Bollen-Stine bootstrap p = .002); therefore, we performed non-parametric bootstrapping procedure (available in AMOS). To scale the factors, the variance of the three factors was set to 1.

#### Gender invariance

To examine gender invariance of MSPSS scores, we conducted multi-group CFA [82?] using the total sample. Measurement invariance was assessed at the configural, metric, and scalar levels [83?]. Configural invariance implies that the latent scales variable(s) and the pattern of loadings of the latent variable(s) on indicators are similar across gender (i.e., the unconstrained latent model should fit the data well in both groups). Metric invariance implies that the magnitude of the loadings is similar across gender; this is tested by comparing two nested models consisting of a baseline model and an invariance model. Lastly, scalar invariance implies that both the item loadings and item intercepts are similar across gender and is examined using the same nested-model comparison strategy as with metric invariance [82?]. Following the recommendations of Cheung and Rensvold (2002) [84?] and Chen (2007) [82?], we accepted ΔCFI ≤ 0.010 and ΔRMSEA ≤ 0.015 or ΔSRMR ≤ 0.010 (0.030 for factorial invariance) as evidence of invariance.

#### Further analyses

Composite reliability in both subsamples was assessed using McDonald’s (1970) ω, with values greater than 0.70 reflecting adequate composite reliability [85?]. McDonald’s ω was selected as a measure of composite reliability because of known problems with the use of Cronbach’s α (e.g., [86?]). The social support total score was considered normally distributed since the skewness (= 0.114) and kurtosis (= -1.086) values varied between ± 2 [87]. Therefore, to assess convergent and criterion-related validity, we examined bivariate correlations between the MSPSS and the resilience and PTG scores using the Pearson test. Based on Cohen (1992) [88], values ≤ 0.10 were considered weak, ~ 0.30 were considered moderate, and ~ 0.50 were considered strong correlations.

## Results

### Participants

Three hundred eighty-seven participants participated in this study, with a mean age of 26.17 ± 11.47 years and 58.4% females. Other descriptive statistics of the sample can be found in Table 1. The description of each item of the MSPSS scale as well as the inter-item correlations can be found in Supplementary Tables 1 and 2 respectively.

**Table 1:** Sociodemographic and other characteristics of the sample (N = 387)

	N (%)
Sex	
Male	161 (41.6%)
Female	226 (58.4%)
Marital status	
Single	311 (80.4%)
Married	76 (19.6%)
Education level	
Secondary or less	66 (17.1%)
University	321 (82.9%)
Region of living	
Urban	294 (76.0%)
Rural	93 (24.0%)

### Confirmatory factor analysis of the MSPSS scale

We tested the one-factor model of the MSPSS scale at first; the fit indices were not good (χ^2^ = 1035.97, df = 54 (*p* < .001), RMSEA = 0.217 (90% CI 0.206, 0.229), SRMR = 0.062, CFI = 0.823, TLI = 0.784).

We then tested the two-factor structures; the fit indices were not good neither for the model where the Family and Significant others factors are merged into one factor [89] (χ^2^ = 586.10, df = 53 (*p* < .001), RMSEA = 0.161 (90% CI 0.150, 0.173), SRMR = 0.042, CFI = 0.904, TLI = 0.881) nor for the model where the Friends and Significant others factors are merged into one factor [90] (χ^2^ = 735.50, df = 53 (*p* < .001), RMSEA = 0.183 (90% CI 0.171, 0.194), SRMR = 0.054, CFI = 0.877, TLI = 0.847).

**Fig. 1:**
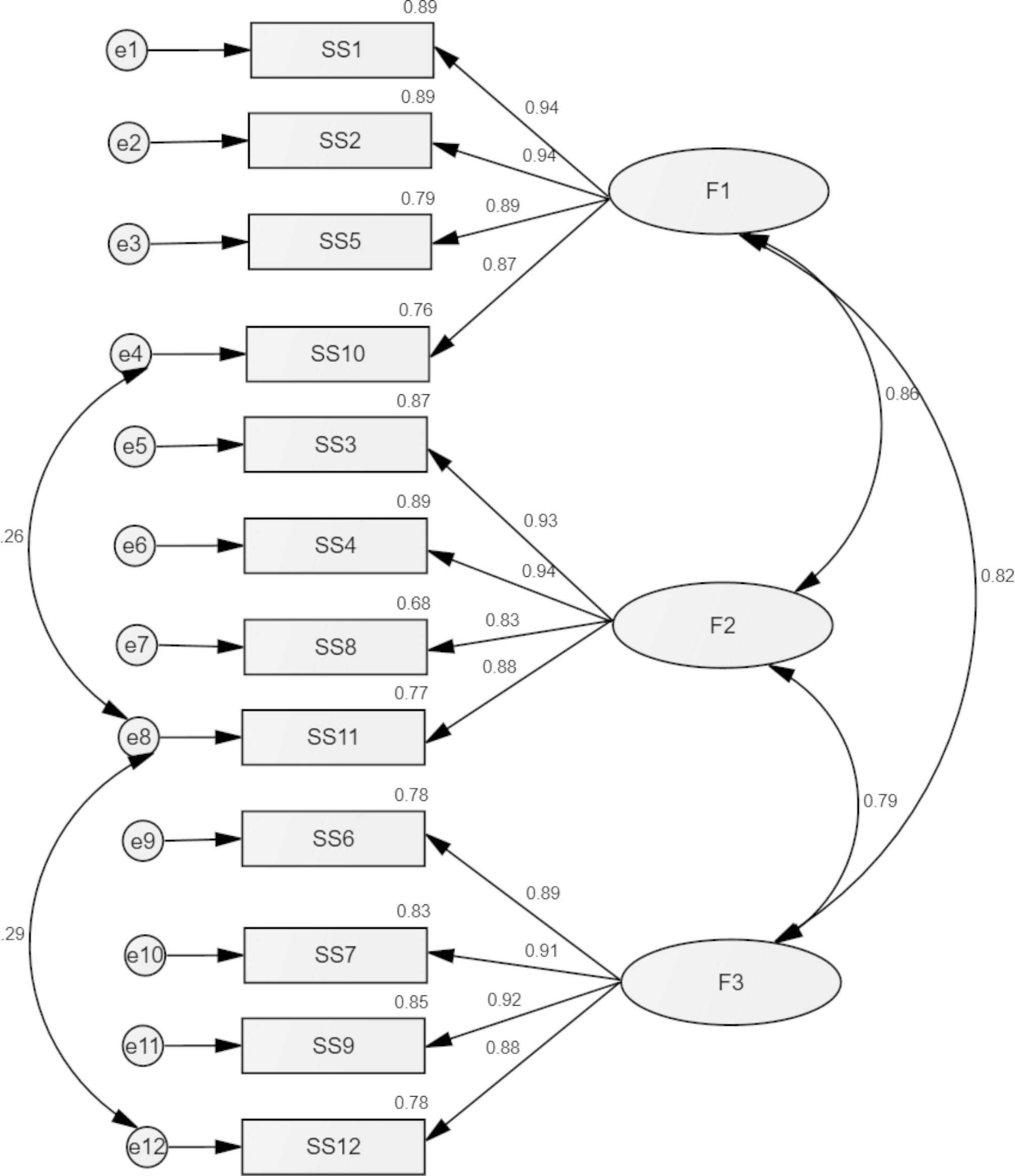
Factor Loadings Derived from the Confirmatory Factor Analysis (CFA) in the total sample. F1 = Social support significant others; F2 = Social support from family; F3 = Social support from friends

CFA indicated that fit of the three-factor model of the MSPSS scale was acceptable: χ^2^ = 246.45, df = 51 (*p* < .001), RMSEA = 0.100 (90% CI 0.087, 0.112), SRMR = 0.034, CFI = 0.965, TLI = 0.954. When adding a correlation between residuals of items 4, 8 and 12, the fit indices improved as follows: χ2 = 192.10, df = 49 (*p* < .001), RMSEA = 0.087 (90% CI 0.074, 0.100), SRMR = 0.032, CFI = 0.974, TLI = 0.965. The standardised estimates of factor loadings were all adequate (see Fig. 1). The convergent validity for this model was good, as AVE = 0.74.

### Gender invariance

As reported in Table 2, all indices suggested that configural, metric, and scalar invariance was supported across gender. Given these results, we computed an independent-samples t-test to examine gender differences in resilience scores. The results showed that there was no statistically significant difference between men and women in all MSPSS dimensions (Table 3).

### Composite reliability

Composite reliability of scores was adequate in the total sample for the MSPSS total scale (ω = 0.97), Significant others (ω = 0.95), Family (ω = 0.94) and Friends (ω = 0.95) subscales.

**Table 2:** Measurement Invariance of the MSPSS Across Gender in the total sample

Model	χ²	df	CFI	RMSEA	SRMR	Model Comparison	Δχ²	ΔCFI	ΔRMSEA	ΔSRMR	Δdf	p
Configural	320.42	102	0.961	0.075	0.035							
Metric	330.59	111	0.961	0.072	0.034	Configural vs. metric	10.17	< 0.001	0.003	0.001	9	0.336
Scalar	333.82	120	0.962	0.068	0.034	Metric vs. scalar	3.23	0.001	0.004	< 0.001	9	0.954

Note. CFI = Comparative fit index; RMSEA = Steiger-Lind root mean square error of approximation; SRMR = Standardised root mean square residual

**Table 3:** Comparison between sexes in terms of the MSPSS total scale and subscales scores in the total sample

	MSPSS total score	MSPSS: Significant others	MSPSS: Family	MSPSS: Friends
Males	52.30 ± 18.24	17.29 ± 6.61	17.84 ± 6.31	17.17 ± 6.40
Females	52.47 ± 19.67	18.15 ± 7.38	17.73 ± 7.17	16.59 ± 6.81
t	0.086	1.18	0.157	0.848
df	385	385	385	385
p	0.932	0.230	0.873	0.397

### Convergent and Criterion-Related Validity

To assess the validity of the scores, we examined bivariate correlations with all other measures included in the present study using the total sample. Higher MSPSS scores and sub-scores were significantly and positively correlated with higher resilience and PTG (Table 4).

**Table 4:** Correlations of the MSPSS total scores and sub-scores with the other measures in the total sample

	Mean ± SD	1	2	3	4	5	6
1. MSPSS total score	52.40 ± 19.06	1					
2. MSPSS: Significant others	17.79 ± 7.07	0.94***	1				
3. MSPSS: Family	17.78 ± 6.82	0.93***	0.83***	1			
4. MSPSS: Friends	16.83 ± 6.64	0.91***	0.78***	0.76***	1		
5. Resilience	23.88 ± 7.29	0.38***	0.36***	0.36***	0.34***	1	
6. Post-traumatic growth	28.04 ± 11.78	0.62***	0.61***	0.60***	0.53***	0.47***	1

*p < .05; **p < .01; ***p < .001; values reflect Pearson correlation coefficients

## Discussion

The purpose of the present study was to rigorously test the psychometric characteristics of the Arabic MSPSS in a non-clinical sample of Lebanese adults; more specifically, to fill the gaps of the first Arabic validation attempt by Merhi et al. [33, 61]. As hypothesized, the Arabic MSPSS maintained excellent psychometric properties in our sample in terms of factor structure, internal consistency, gender invariance and convergent validity. The findings, therefore, provided strong evidence that the Arabic version of the MSPSS is a suitable self-report measure of social support for use in research and clinical practice in Arab settings.

Our analyses indicated an adequate fit indices for the three-factor model, which is in line with the original development study [17], and several other previous language adaptations in non-clinical samples [91–93]. Given that perceptions of social networks are shaped by culture [94–96], MSPSS may show inconsistent structural validity across many various cultural settings. For instance, certain previous validation studies, mainly in Asian countries, yielded a unidimensional factor structure (e.g., [27, 28, 97–101]). Other psychometric studies reported a 2-factor structure, with either Family and Significant others [89] or Friends and Significant others [90] subscales merging into one factor. Hence the importance of performing CFA in addition to EFA which, when used alone, could result in inappropriate conclusions [102]. Beyond factor structure, our results demonstrated that the Arabic MSPSS and its subscales have a high internal consistency with McDonald’s ω values between 0.94 and 0.97 [103]. A good internal consistency of the MSPSS has been consistently supported through high Cronbach’s alphas coefficients in the original English [17] as well as other language versions [26, 27, 32, 104, 105]. While prior evidence has shown that Cronbach’s alpha is inappropriate in estimating the internal consistency of multidimensional instruments [106], and that McDonald’s ω is particularly more advantageous [106], very few studies used McDonald’s ω when assessing the internal consistency of MSPSS [41, 107].

Both genders exhibited no significant difference in all MSPSS dimensions. Our analyses also established measurement invariance across gender at the configural, metric, and scalar levels. These findings support the appropriateness of the MSPSS in measuring identical constructs with the same 3-factor structure across genders, and its usefulness in comparing the mean scores of the three social support subscales (Family, friends, and the significant other) between males and females. In the original validation by Zimet et al. [17], male undergraduates displayed lower perceived support from friends and the significant other, as well as lower total support than females. Other studies documented mixed patterns of gender differences (i.e., greater support in all dimensions among females [20], more support from family and friends among females [24], greater support from family among males [108], no gender difference [109]). These discrepancies may be explained by variations in socialized gender-roles across societies and cultures; which calls for further cross-cultural research on gender differences in perceived social support to confirm our assumptions. Finally, convergent validity was supported by showing that all three MSPSS sub-scores and total score correlated significantly and positively with resilience and PTG scores. These expected patterns of associations are in agreement with previous studies in which social support has consistently been found to be significantly related to both resilience [110] and PTG [111, 112] constructs. Resilience has been suggested to enable more positive perceptions of resources, which in turn acts as a stress buffer [113, 114]. Similarly, greater interpersonal resources were found to be amongst the positive changes undertaken by individuals after trauma [115].

### Study limitations

First, our sample lacks diversity, comprised exclusively of Arabic-speaking Lebanese people. Future research should consider validating the MSPSS in other Arab countries and communities. Second, we adopted a cross-sectional design and a self-report method to collect our data; longitudinal validation studies are still required to assess predictive validity. Third, while some fit indices did not show adequate values (such as RMSEA value), some psychometric properties were not examined in the context of the present study, such as responsiveness and test-retest reliability, and should be subject of additional research to confirm temporal stability of the MSPSS. The forward and backward translations should have been made by at least two independent translators each time [116]. The number of participants in the pilot study might be small [117]. A selection bias might be present because of the snowball sampling technique method used to collect the data and the unknown refusal rate. Information bias might be present since the answers were self-reported by participants and not evaluated by a healthcare professional. Finally, due to the web-based and self-report nature of the data collection, we were not able to exclude respondents who did not understand the questionnaire due to cognitive impairment or other diseases.

## Conclusion

We believe that this study will add to the body of literature on perceived social support by providing a psychometrically sound Arabic version of the MSPSS, with good factorial and convergent validity, as well as high internal consistency and gender invariance. Although further cross-cultural validations involving other Arab countries and communities are still needed, we preliminarily suggest that this scale is applicable to the broad Arabic-speaking people for the measurement of perceived social support in clinical and research contexts.

## Data Availability

All data generated or analyzed during this study are not publicly available due the restrictions from the ethics committee, but are available upon a reasonable request from the corresponding author (SH).
